# Cardiopulmonary Bypass and Oxidative Stress

**DOI:** 10.1155/2015/189863

**Published:** 2015-02-04

**Authors:** Mustafa Zakkar, Gustavo Guida, M-Saadeh Suleiman, Gianni D. Angelini

**Affiliations:** Bristol Royal Infirmary, Level 7, Upper Maudlin Street, Bristol BS2 8HW, UK

## Abstract

The development of the cardiopulmonary bypass (CPB) revolutionized cardiac surgery and contributed immensely to improved patients outcomes. CPB is associated with the activation of different coagulation, proinflammatory, survival cascades and altered redox state. Haemolysis, ischaemia, and perfusion injury and neutrophils activation during CPB play a pivotal role in oxidative stress and the associated activation of proinflammatory and proapoptotic signalling pathways which can affect the function and recovery of multiple organs such as the myocardium, lungs, and kidneys and influence clinical outcomes. The administration of agents with antioxidant properties during surgery either intravenously or in the cardioplegia solution may reduce ROS burst and oxidative stress during CPB. Alternatively, the use of modified circuits such as minibypass can modify both proinflammatory responses and oxidative stress.

## 1. Introduction

The development of the cardiopulmonary bypass (CPB) revolutionized cardiac surgery and contributed immensely to improved patients outcomes [1, 2].

**Figure 1:**
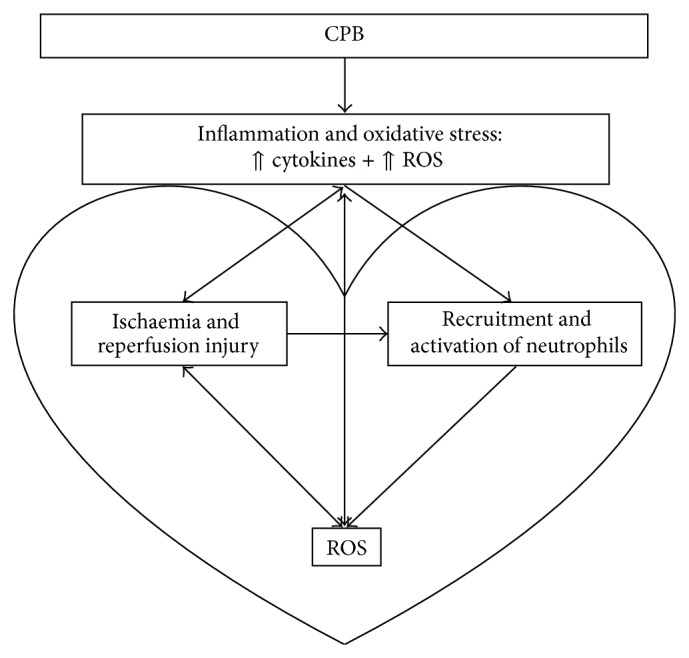
Schematic overview of inflammatory and oxidative stress response during cardiopulmonary bypass.

It is accepted that CPB exposes patients to a complex set of nonphysiological conditions during which organs are subjected to severe functional alterations. CPB is associated with the activation of different coagulation, proinflammatory, survival cascades and altered redox state [3–5] (Figure 1). Despite significant refinements over the years, oxidative stress and inflammation remain major concerns when using CPB [6, 7].

## 2. Reactive Oxygen Species

Free radicals are molecules with unpaired electrons making them highly reactive. They can be derived from oxygen, nitrogen, or sulfur molecules. Free radicals derived from oxygen are usually called reactive oxygen species (ROS) [8, 9]. The main forms of cardiac ROS are superoxide (O_2_^−•^), hydrogen peroxide (H_2_O_2_), hydroxyl radicals (^•^OH), and peroxynitrite (ONOO^−^). Although hydrogen peroxide is not a free radical it is considered a ROS due to its highly reactive nature.

ROS can be formed as a natural byproduct of normal metabolism of oxygen as aerobic metabolism results in the production of ROS in the mitochondria during ATP formation. Nonetheless, cells are normally able to shield themselves against ROS through different defense mechanisms that include enzymatic and free radical scavenging activities to neutralize these radicals resulting in a balance (redox state) between ROS production and cells ability to detoxify ROS or to repair any subsequent damage. During periods of stress, however, ROS levels can increase drastically leading to substantial damage to many cellular molecules such as lipids, proteins, and DNA [8–10].

Redox signalling occurs as a response to changes in the levels of ROS and the mitochondria appear to be of pivotal importance for this signalling due to its role in metabolism, the continuous flux of O_2_^•−^ and oxygen sensing. Redox signalling is involved in multiple processes such as homoeostasis, stress response pathways, and cardiac remodeling and fibrosis [8, 10–12].

## 3. Oxidative Stress, Inflammation, and Cardiopulmonary Bypass

The presence of atherosclerotic coronary artery disease requiring intervention is associated with evidence of oxidative stress and inflammation prior to surgery which can be markedly accentuated during CPB [13]. Furthermore, patients undergoing cardiac surgery tend to have other coexisting morbidities such as diabetes and renal and lung diseases which are associated with abnormal redox state and oxidative stress.

**Figure 2:**
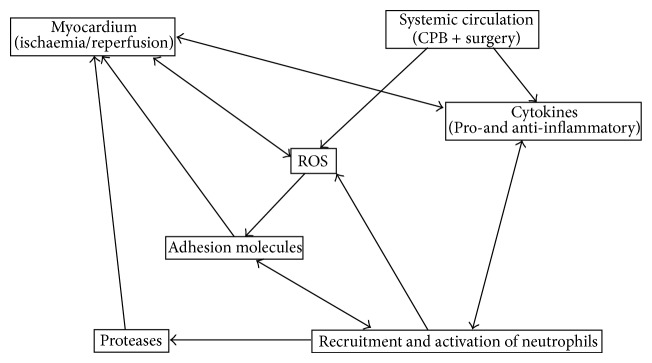
Main triggers of inflammatory response during cardiopulmonary bypass.

CPB initiates multiple processes that impact both cellular and noncellular contents of blood. The repeated passage of blood through the nonendothelialised extracorporeal circuit triggers the activation of polymorphonuclear leukocytes (mainly neutrophils) which are believed to be a prime source of ROS during cardiac surgery. Activation of neutrophils during CPB is evident by the loss of L-selectin and the upregulation of CD11b/CD18 (Mac-1) [14–17] (Figure 2).

During CPB, ischaemic injury occurs when the blood supply to tissue is suboptimal and accompanied by cellular adenosine triphosphate depletion due to its degradation by hypoxanthine [7, 18]. Hypoxanthine is normally oxidized by the enzyme xanthine dehydrogenase to xanthine using nicotinamide adenine dinucleotide (NAD) in a reaction converting NAD to nicotinamide adenine dinucleotide hydrogenase (NADH), conversely; xanthine dehydrogenase is converted to xanthine oxidase during periods of ischaemia. Furthermore, anaerobic metabolism results in the production of lactic acid and altered cellular homeostasis with the loss of ion gradients across cell membranes [7, 16–19]. Reperfusion after a period of ischaemia plays a pivotal role in oxidative stress by initiating a series of biochemical events that result in the generation of excessive amount of ROS. Reduction of oxygen leads to the production of the superoxide anion, which is able to penetrate through cell membranes where it is converted into other more toxic oxygen species. Therefore, the dismutase reaction (catalyzed by superoxide dismutase) leads to the conversion of the superoxide anion into hydrogen peroxide. This can lead to the production of hypochlorous acid by the action of MPO, or through interaction with iron salts, in the Haber-Weiss reaction to generate the highly toxic hydroxyl radical. The toxicity of the hydroxyl radical results from its ability to take electrons from a wide range of molecules, leading to the formation of a new radical that can continue the reaction [16, 17, 19].

ROS can modulate signalling proteins activity by nitrosylation, carbonylation, disulphide bond formation, and glutathionylation triggering the activation of proinflammatory and proapoptotic signalling pathways such as MAPK and NF-*κ*B.

Superoxide radicals (O_2_^−^) are generated rapidly in EC by the NADPH oxidase complex in response to TNFR [20]. Application of antioxidants to EC or the overexpression of HO-1 can inhibit the induction of E-Selectin and VCAM-1 whereas ICAM-1 is relatively refractory [21, 22]. MAPK activities are tightly regulated by redox. Antioxidants shorten the kinetics of MAPK activation following treatment with TNF*α*, suggesting that endogenous ROS are essential for prolonged MAPK activity and the underlying mechanism may involve suppression of negative regulators of MAPK by ROS [23]. For example, in resting cells, thioredoxin suppresses MAPK signalling pathways by interacting with the N-terminal portion of ASK1 to inhibit its catalytic activity [24]. This interaction is disrupted by ROS which alters the structure of thioredoxin by triggering the formation of intramolecular disulphides. Thus the prolonged activation of MAPK in response to proinflammatory signalling relies on disruption of thioredoxin-ASK1 interaction by ROS [24].

Activation of NF-*κ*B under oxidative stress has been reported in a number of inflammatory conditions [25–27]. Oxidative stress-induced LDAs and their GS-conjugates play a major role in the mediation of NF-*κ*B-induced inflammatory signals via PLC/PKC/IKK/MAPK pathways. Furthermore, ROS can act as toxic messengers that activate NF-*κ*B and affect the cellular functions of growth factors, cytokines, and other molecules [28, 29].

CPB and ischaemia and reperfusion injury during surgery can cause substantial myocardial stress leading to the generation of proinflammatory mediators and ROS resulting in damage to proteins, lipids, and DNA which impact on postoperative cardiac functional and outcomes [13].

Ischaemia leads to reduction in mitochondria energy production due to the lack of oxygen and nutrients. This is followed by fall in ATP, decrease in intracellular pH, and a rise in intracellular concentrations of Na^+^ and Ca^2+^ [7, 18] Furthermore, cardiac myocytes exposed to ischaemia react by producing proinflammatory cytokines and the consequent activation of leukocyte adhesion cascade allowing neutrophils to accumulate in the myocardium, adhere to the myocytes, and release ROS and other proteolytic enzymes. Moreover, reperfusion can lead to irreversible myocardial damage due to mitochondrial dysfunction driven by cytosolic Ca^2+^ loading and generation of ROS [7, 30, 31]. ROS can also stimulate the opening of the mitochondrial permeability transition pore (mPTP) leading to further ROS production and generating a positive feedback loop of ROS formation and mPTP opening [32–35]. The opening of mPTP can also result in mitochondrial swelling, mitochondrial membrane damage, and cell death via either apoptosis or necrosis [35, 36].

CPB exposes RBCs to abnormal settings resulting in integrity and function alteration. Shear stress forces generated by the CPB pump cause mechanical damage to the RBCs and induce ionic pump changes at the cell surface and an abnormal accumulation of intracellular cations [37–39]. This reduces RBC deformability which is important for maintaining normal microcirculation by making them more rigid and fragile. Furthermore, and as a consequence of membrane distortion, RBC become vulnerable to the membrane attack complex (MAC) generated from the activation of complement, leading to haemoglobin leak, thus substantially increasing the concentration of free Hb which can act as a peroxidase in the presence of H_2_O_2_ [37, 39–41].

Blood transfusion during or postsurgery is associated with increased oxidative stress due to the use of stored blood which has decreased antioxidant properties. Blood storage will lead to changes in erythrocytes (storage defect) which include depletion of adenosine triphosphate and 2,3-diphosphoglycerate, alterations in nitric oxide-mediated functions, and increased lipid peroxidation [42, 43]. Moreover; erythrocyte membrane changes make them less deformable and more fragile with an increased tendency toward progressive haemolysis leading to the accumulation of free haemoglobin and iron in the circulation which are redox active and prooxidant as discussed previously.

Increased ROS production and, more specifically, increased superoxide anion production derived from the atrial nicotinamide adenine dinucleotide phosphate oxidase are independently associated with a higher risk of POAF supporting an association between ROS in human atrium and POAF [44, 45]. Furthermore, atrial tissue from patients in whom POAF developed displayed an upregulation of mitochondrial MnSOD activity and an increased sensitivity of mPTP opening [45, 46].

It is recognised that cardiac surgery using CPB is associated with a wide spectrum of acute lung injury which can manifest in its most severe form as acute respiratory distress syndrome (ARDS). ARDS is rare after surgery; however it can have significant impact on morbidity and mortality. It has been thought that complement and neutrophils activation alongside red blood cell damage and reoxygenation injury in lung tissue are responsible for altering the redox status towards oxidative stress conditions. Bronchoalveolar lavage and plasma analysis of patients with ARDS after cardiac surgery has shown evidence of severe oxidative stress including the presence of high levels of chlorotyrosine, nitrotyrosine, and orthotyrosine [47–49].

Acute kidney injury (AKI) as a complication of cardiac surgery using CPB can be triggered by many elements including ischaemia and reperfusion injury, altered blood flow patterns, haemolysis, and blood transfusion [50, 51]. Haemolysis associated with increased levels of free haemoglobin in conjunction with increased levels of plasma myoglobin has been shown to be independent predictors of AKI after CPB due to their prooxidant properties. Ischaemia and reperfusion injury with the associated depletion of energy in renal epithelial cells causes mitochondrial dysfunction, the release of ROS, and the activation of proinflammatory signalling pathways (MAPK and NF-*κ*B) that can cause disruption of the cytoskeleton and lead to cell tubular damage [52–55]. Furthermore, these events taking place in the kidney will result in active neutrophil sequestration to renal tissue and more ROS generation [53, 55].

## 4. Intervention to Reduce Oxidative Stress during CPB

### 4.1. Antioxidant Supplements

The effects of ROS are counteracted under physiological conditions by antioxidants which are molecules that are capable of neutralizing free radicals by accepting or donating electron(s) thereby eliminating the unpaired condition of the radical. The antioxidant molecules may directly react with the reactive radicals and destroy them, while they may become new free radicals which are less active, longer-lived, and less dangerous than those radicals they have neutralized. They may be neutralized by other antioxidants or other mechanisms to terminate their radical status.

The use of additive antioxidants such as propofol, L-arginine, and N-acetyl-cysteine (NAC) during CPB as intravenous infusion or mixed with cardioplegia can be an appropriate strategy to counteract the impact of ROS.

Propofol (2,6-diisopropylphenol) is a commonly used agent in cardiac surgery for both induction and maintenance of anaesthesia. The phenolic hydroxyl group included in Propofol's structure is similar to vitamin E which is known to be a natural antioxidant. Multiple* in vitro* and* in vivo* studies have demonstrated significant antioxidant effects of propofol [56–58]. It has been reported to inhibit lipid peroxidation to protect cells against oxidative stress and to increase the antioxidant capacity of plasma in humans [59]. Propofol can react with peroxynitrite, leading to the formation of a propofol-derived phenoxyl radical and it therefore can act as a peroxynitrite scavenger. Furthermore, propofol can protect against peroxynitrite-mediated cytotoxicity, DNA ladderization, and apoptosis [59–61]. Moreover propofol administrated intravenously can attenuate the activation of NF-*κ*B [62] which has a pivotal role in oxidative stress and inflammatory responses activated during ischemia/reperfusion.

Animal studies demonstrated that propofol increases the antioxidant capacity of the liver, kidney, heart, and lung by decreasing t-BHP-induced TBARS formation in an ischaemia and reperfusion model [56–58, 63]. During CPB, RBC antioxidant capacity as measured by MDA production is enhanced and maintained with the administration of intravenous propofol. Furthermore, the administration of a large dose of propofol during CPB attenuates postoperative myocardial cellular damage in patients undergoing CABG [64]. We have previously demonstrated in an animal model that propofol protects isolated perfused rat hearts from ischaemia/reperfusion injury through the preservation of mitochondrial function during reperfusion by inhibiting mitochondrial permeability transition pore opening [65]. Moreover, in another animal study, we demonstrated that propofol infusion during CPB attenuated the changes in myocardial tissue levels of adenine nucleotides, lactate, and amino acids during ischaemia and reduced cardiac troponin I release on reperfusion [66]. Furthermore, the permeability transition in mitochondria isolated from hearts treated with propofol was less sensitive to [Ca^2+^] than control mitochondria. The result of a randomised trial designed to address the effect of adding propofol to cardioplegia solution in patients undergoing cardiac surgery will report shortly [67].

L-Arginine is an amino acid that can play an important role in immune function and vascular homeostasis as a precursor for the synthesis of nitric oxide (NO). It is recognised that cardiac surgical patients have arginine/nitric oxide pathway impairment evident by increased levels of nitric oxide inhibitor asymmetric dimethylarginine [68, 69]. The use of oral L-arginine supplements in patients undergoing heart transplant resulted in reduced vascular endothelial cells dysfunction and decreased serum H_2_O_2_ production suggesting an increase in bioavailability of NO [70]. Such changes were translated clinically by improved exercise capacity of postsurgery [71]. When L-arginine was added to cardioplegia solutions in patients undergoing CABG, it was associated with higher Myocardial O_2_ uptake and reduced malondialdehyde (MDA) extraction [72, 73]. In patients with impaired LV function, L-arginine cardioplegia decreases biochemical markers of myocardial damage and oxidative stress and resulted in increased superoxide dismutase activity [74].

NAC is another effective scavenger of free radicals with neutrophil aggregation inhibitory properties. When NAC is used as intravenous infusion during CABG surgery, it was noted that luminol, H_2_O_2_, HOCl-, and lucigenin levels were significantly lower than the control group. Furthermore, tumour necrosis factor-alpha levels were significantly decreased as a result of NAC administration [75, 76]. Moreover, MDA levels were significantly lower in the NAC enriched group during the reperfusion period [75]. When adding NAC to cardioplegia solution, it was noted that it can significantly reduce serum MDA, glutathione, catalase, SOD, glutathione peroxidase, and glutathione reductase [77–79].

Levosimendan is a calcium sensitizer that enhances myocardial contractility without increasing myocardial oxygen. Its function involves the activation of mitochondria ATP-sensitive potassium (mito-KATP) channels and production of eNOS-dependent NO through Akt and MAPK [80, 81].

KATP channels belong to the ATP-binding cassette transporter superfamily. Two KATP channel subtypes coexist in the myocardium, with one subtype located in the sarcolemma (sarc-KATP) membrane and the other in the inner membrane of the mito-KATP [82]. Under homeostatic conditions KATP channels are closed. However, under conditions of ischemia/reperfusion, KATP channels are activated allowing a net influx of K^+^ ions into the matrix and preventing Ca^2+^ accumulation in the matrix, thus blunting the opening of mPTP and its deleterious effects (as discussed above). Furthermore, the activation of mito-KATP can protect cells from damage induced by ROS and reduce cell apoptosis [83–86]. Clinical studies in patients undergoing cardiac surgery showed that Levosimendan significantly enhanced primary weaning from CPB compared with placebo in patients undergoing CABG. Moreover, the need for additional inotropic or mechanical therapy was decreased especially in patients with blunted ventricular function [87–90]. Furthermore, a meta-analysis which was of randomised studies including 529 patients in 5 trials suggested that Levosimendan might reduce renal injury in adult patients undergoing cardiac surgery [91].

Recent years have witnessed the use of mitochondrial targeted antioxidants such as Mitoquinone mesylate (MitoQ). MitoQ is made up of a lipophilic triphenylphosphonium (TPP) cation covalently attached to a ubiquinone antioxidant moiety. The positive charge on the TPP cation means that it is rapidly and extensively taken up by mitochondria due to the large membrane potential across the mitochondrial inner membrane. Within mitochondria, the ubiquinone moiety of MitoQ is rapidly reduced by complex II of the mitochondrial respiratory chain to the active antioxidant ubiquinol form and, after detoxifying a ROS, it is converted to the ubiquinone form which is then rapidly recycled back to the active antioxidant [92, 93].

MitoQ has been used in a wide range of animal studies and demonstrated efficacy in preventing mitochondrial oxidative damage in the heart, liver, kidney, and brain [92, 94–97]. Furthermore, MitoQ has been shown to be protective against liver damage in hepatitis C patients [98]. The combination of strong evidence indicating a pivotal role of mitochondrial damage in CPB related oxidative stress and data from animal studies showing that MitoQ prevents such damage in the heart provides a compelling case for evaluating the effectiveness of MitoQ in protecting the myocardium as an additive to cardioplegia solution during cardiac surgery to counteract the effect of CPB.

### 4.2. Mini-CPB

The impact of surface contact activation, air-fluid interface, and cell damage by cardiotomy suction associated with conventional bypass on the activation of proinflammatory and survival cascades has led to the development of minimised cardiopulmonary bypass circuit (mini-CPB). The design is a closed CPB system characterised by reduced surface area and thus priming volume, elimination of cardiotomy suction, and prevention of air-blood contact. The use of mini-CPB is associated with delayed or reduced secretion of different proinflammatory cytokines, attenuated complement activation, and blunted leukocytes activation compared to conventional circuit [99, 100].

The utilization of mini-CPB is associated with significant reduction in red blood cell damage (as measured by free Hb), activation of coagulation cascades, and blunted fibrinolytic and proinflammatory activities. Furthermore, serum concentration of MDA and allantoin/urate ratio as markers of oxidative stress tend to be reduced in patients undergoing surgery using mini-CPB when compared to conventional circuit [101, 102].

## 5. Conclusions

Cardiopulmonary bypass, although not perfect, remains an essential part of cardiac surgery. The utilization of CPB is associated with the production of ROS and oxidative stress.

Haemolysis, ischaemia, and perfusion injury and neutrophils activation during CPB play a pivotal role in oxidative stress and the associated activation of proinflammatory and proapoptotic signalling pathways which can affect the function and recovery of multiple organs such as the myocardium, lungs, and kidneys and influence clinical outcomes.

The administration of agents with antioxidant properties during surgery either intravenously or in the cardioplegia solution may reduce ROS burst and oxidative stress during CPB. Alternatively, the use of modified circuits such as minibypass can modify both proinflammatory responses and oxidative stress. More in-depth research and adequately powered randomised clinical studies with strict CPB protocols are still required.
